# Surface properties of dental zirconia ceramics affected by ultrasonic scaling and low-temperature degradation

**DOI:** 10.1371/journal.pone.0203849

**Published:** 2018-09-13

**Authors:** Kosuke Nakazawa, Keisuke Nakamura, Akio Harada, Midori Shirato, Ryoichi Inagaki, Ulf Örtengren, Taro Kanno, Yoshimi Niwano, Hiroshi Egusa

**Affiliations:** 1 Division of Molecular and Regenerative Prosthodontics, Tohoku University Graduate School of Dentistry, Sendai, Japan; 2 Department of Advanced Free Radical Science, Tohoku University Graduate School of Dentistry, Sendai, Japan; 3 Tohoku University School for Dental Laboratory Technicians, Sendai, Japan; 4 Department of Cariology, Institute of Odontology, Sahlgrenska Academy, University of Gothenburg, Gothenburg, Sweden; 5 Department of Clinical Dentistry/Faculty of Health Sciences, The Arctic University of Norway, Tromsø, Norway; 6 Faculty of Nursing, Shumei University, Chiba, Japan; Institute of Materials Science, GERMANY

## Abstract

Zirconia (3Y-TZP) dental prostheses are widely used in clinical dentistry. However, the effect of ultrasonic scaling performed as a part of professional tooth cleaning on 3Y-TZP dental prostheses, especially in conjunction with low-temperature degradation (LTD), has not been fully investigated. The present study aimed to evaluate the influence of ultrasonic scaling and LTD on the surface properties of 3Y-TZP in relation to bacterial adhesion on the treated surface. 3Y-TZP specimens (4 × 4 × 2 mm) were polished and then subjected to autoclaving at 134°C for 100 h to induce LTD, followed by 10 rounds of ultrasonic scaling using a steel scaler tip for 1 min each. Surface roughness, crystalline structure, wettability, and hardness were analyzed by optical interferometry, X-ray diffraction analysis, contact angle measurement, and nano-indentation technique, respectively. Subsequently, bacterial adhesion onto the treated 3Y-TZP surface was evaluated using *Streptococcus mitis* and *S*. *oralis*. The results demonstrated that the combination of ultrasonic scaling and LTD significantly increased the Sa value (surface roughness parameter) of the polished 3Y-TZP surface from 1.6 nm to 117 nm. LTD affected the crystalline structure, causing phase transformation from the tetragonal to the monoclinic phase, and decreased both the contact angle and surface hardness. However, bacterial adhesion was not influenced by these changes in surface properties. The present study suggests that ultrasonic scaling may be acceptable for debridement of 3Y-TZP dental prostheses because it did not facilitate bacterial adhesion even in the combination with LTD, although it did cause slight roughening of the surface.

## Introduction

Zirconia is widely used in dentistry owing to its excellent mechanical properties as well as the development of computer-aided design/computer-aided manufacturing (CAD/CAM) technology [1, 2]. Zirconia dental prostheses include frameworks for all-ceramic fixed dental prostheses (FDPs), dental implant abutments, and monolithic restorations. According to systematic reviews, the cumulative 5-year survival rate for zirconia-based FDPs is over 90% [3–6], which is comparable to that of metal-ceramic FDPs.

The longevity of dental prostheses largely depends not only on the quality of the prosthetic treatment but also on the plaque control continuously performed by patients and dental professionals to prevent development and recurrence of dental caries and/or periodontal disease [7, 8]. Professional tooth cleaning complements self-performed plaque control by providing cleaning in difficult-to-reach areas, such as the subgingival area. In addition, professional cleaning, using instruments such as ultrasonic scalers, is performed to dislodge mineralized deposits (i.e. dental calculus) that cannot be removed in self-performed plaque control by patients. An ultrasonic scaler is a machine-driven instrument that vibrates a scaler tip at around 30 kHz. The scaler vibration can mechanically remove dental plaque and calculus from tooth surfaces. Since the efficacy of subgingival debridement using ultrasonic scalers has been shown to be comparable to that of hand scalers, but requiring less time [9], ultrasonic scaling has become widely used for professional tooth cleaning. Thus, dental prostheses, including zirconia prostheses, may be subjected to ultrasonic scaling. This raises concerns regarding the damage to the zirconia surface, especially when the ultrasonic scaling is repeatedly performed at regular intervals after prosthetic treatment. The surface properties (e.g. surface roughness, surface free energy, and chemical conditions of the topmost surface) of zirconia play an important role in achieving appropriate biocompatibility and in reducing bacterial adhesion [10, 11]. Ultrasonic scaling may mechanically alter the surface properties. A previous *in vitro* study demonstrated that ultrasonic scaling did not increase the surface roughness of zirconia, and as such, did not facilitate bacterial adhesion to the treated surface [12]. However, ultrasonic scaling was performed over only five strokes at three locations on the specimen in that study. Additional studies are necessary to evaluate the influence of repeated ultrasonic scaling.

Among zirconia-containing ceramics, 3 mol% yttria-stabilized tetragonal zirconia polycrystal (3Y-TZP) is the most widely used in dentistry [13, 14]. 3Y-TZP is mainly composed of the metastable tetragonal phase. When exposed to mechanical stress, the metastable tetragonal phase is transformed into the monoclinic phase with volume expansion of the grains, resulting in generation of compressive stress that contributes to resistance against crack propagation [15]. This phenomenon is known as stress-induced transformation toughening [16]. The metastable tetragonal phase also spontaneously transforms into the monoclinic phase in a humid atmosphere without mechanical stress, which is referred to as low-temperature degradation (LTD) or aging [17]. Although this process occurs very slowly at body temperature, it may affect the long-term performance of 3Y-TZP dental prostheses. When LTD progresses, the phase transformation decreases the mechanical strength of 3Y-TZP differently from stress-induced transformation toughening, because the phase transformation occurs on the entire surface of the dental prostheses and is accompanied by microcrack generation due to the volume expansion of the grains [18–20]. This degraded surface may show greater damage from the instrumentation performed for professional plaque control. In addition, LTD can itself also cause surface coarsening via generation of microcracks and grain pull-out [21]. However, no study has fully investigated how LTD affects the resistance of 3Y-TZP dental prostheses to repeated instrumentations and the subsequent dental plaque formation.

Therefore, we hypothesized that repeated ultrasonic scaling in combination with LTD would increase the surface roughness of 3Y-TZP, thereby accelerating bacterial adhesion on the surface. The purpose of the present study was to test this hypothesis by examining the influence of ultrasonic scaling and LTD on surface roughness, crystalline structure, wettability, and hardness of 3Y-TZP, in relation to the adhesion of oral streptococci on the treated surface.

## Materials and methods

### Preparation of specimens

Forty-two specimens of 3Y-TZP with dimensions of 5 × 5 × 2.5 mm were cut from pre-sintered zirconia blocks (Lava Plus Zirconia; 3M/ESPE, St. Paul, MN, USA) using an Isomet 4000 (Buehler, Lake Bluff, IL, USA). The specimens were sintered at 1450°C for 2 h (Lava Furnace 200; 3M/ESPE) according to the manufacturer’s instructions. After sintering, the specimens had dimensions of 4 × 4 × 2 mm with an error range of ±0.1 mm. One side of each specimen was polished using an automatic grinder-polisher (MetaServ 250; Buehler) with 9-, 6-, and 1-μm diamond suspensions (MetaDi Supreme, Buehler). Thirty-six specimens were used in a series of nondestructive tests (i.e., analyses of surface roughness, crystalline structure, wettability, and bacterial adhesion). They were divided into six groups (n = 6); (1) untreated (UT), (2) ultrasonic scaling using a plastic scaler tip (US-P), (3) ultrasonic scaling using a steel scaler tip (US-S), (4) autoclaved to induce LTD without additional treatment (LTD-UT), (5) autoclaving followed by US-P (LTD-US-P), and (6) autoclaving followed by US-S (LTD-US-S). Autoclaving was performed under the conditions described below. The remaining 6 specimens were used for analysis of the cross-sectional surface by scanning electron microscopy (SEM). In addition, 12 rectangular plate specimens (final dimensions: 8 × 4 × 2 mm) were prepared for a nano-indentation hardness test with the same process described above.

### Surface treatment

The specimens in the LTD group were subjected to autoclaving at 134°C and at 0.2 MPa for 100 h in an autoclave (LSX-300; Tomy Seiko, Tokyo, Japan). Autoclaving-induced LTD was employed as an accelerated aging test. The temperature for autoclaving and treatment time were determined according to ISO standard 13356:2008 [22] and previous studies [19, 20], respectively.

An operator (dentist) conducted a calibration procedure to perform ultrasonic scaling with a pressure of approximately 0.2 N, using an electronic balance. An ultrasonic scaler (miniMaster Piezon LED; EMS, Nyon, Switzerland) was used at a power setting of 70%, which is the setting used for subgingival debridement in periodontal therapy. Pure water produced by a water purification system (Synergy UV; Millipore, Darmstadt, Germany) was used as coolant, at a flow rate of 50 mL/min. The scaling was performed using either a plastic scaler tip made of polyether ether ketone (CLiP&Insert-i; Scorpion, Romagnat, France) or a stainless-steel scaler tip (Piezo Tip-P; EMS). The former and the latter scaler tips were replaced with new tips after 3 and 30 min of use, respectively. The lateral surface of the scaler tip was contacted to the polished surface of the specimens, as in the case of ultrasonic scaling of the tooth surface. Ultrasonic scaling was performed over the entire surface of the specimen as equally as possible for 1 min and repeated 10 times. After scaling, the specimens were ultrasonically cleaned in pure water for 5 min.

### Evaluation of surface properties

#### Surface roughness

Surface roughness analysis was performed using an optical interferometer (TalySurf CCI HD-XL; Taylor Hobson, Leicester, UK) with a 50× objective lens. The resolution was set at 1024 × 1024 pixels. The acquired image was leveled and processed using a Gaussian filter with a cutoff value of 80 μm to compute the surface roughness parameters, including Sa (arithmetic mean height), Sq (root mean square height), Sp (maximum peak height), Sv (maximum valley depth), and Sz (distance from peak to valley). Since the ultrasonic scaling was performed manually, possibly generating an uneven surface roughness, measurements were performed at four sites per specimen, chosen from the upper right, upper left, lower right, and lower left areas. The mean value was regarded as the representative value of the specimen. All specimens used in the nondestructive test were subjected to surface roughness analysis after autoclaving of the specimens in the LTD group (n = 18 for each non-LTD group and LTD group) and after ultrasonic scaling (n = 6 for each group).

#### Crystalline structure

The crystalline structure of the specimens was analyzed using a *θ–2θ* X-ray diffractometer (XRD; SmartLab; Rigaku, Tokyo, Japan). From each treatment group, 3 specimens were randomly selected for XRD analysis. Diffractograms with Cu-Kα radiation were obtained from 27° to 33° at a scan speed of 20°/min and a step size of 0.01°. The monoclinic volume fraction, *Vm*, was calculated using the Garvie and Nicholson method [23], modified by Toraya [24],

Vm=1.311×Xm/(1+0.311×Xm),

withXm=[Im(‑111)+Im(111)]/[Im(‑111)+Im(111)+It(101)]

where *It* and *Im* represent the integrated intensity of the tetragonal (101) and monoclinic (111) and (−111) peaks. The integrated intensity of each peak was calculated using the device software (PDXL; Rigaku). The monoclinic phase fraction was expressed as the percentage of the tetragonal phase.

In addition, to analyze the depth of the monoclinic layer, the cross-sectional surface of specimens was analyzed using an SEM (SU5000; Hitachi, Tokyo, Japan) with a detector for electron backscattered diffraction analysis (EBSD; EDAX Pegasus EDS/EBSP; Ametek, Berwyn, PA, USA). Half of the specimens (n = 3) prepared for this analysis were subjected to autoclaving under the same conditions as described above, and the remaining 3 specimens were used without autoclaving. After autoclaving, the specimen was cut at the center, and then embedded in epoxy resin to evaluate the cross-sectional surface. The cross-sectional surface was polished as described above, which was followed by further polishing using colloidal silica suspension (MasterMet, Buehler). Backscattered electron (BSE) images of the cross-sectional surface were obtained under low vacuum condition (30 Pa) for measurement of the transformed depth. Subsequently, the EBSD detector was inserted into the specimen chamber, and the specimen was tilted 70° so that the electron diffraction could be detected. Based on the diffraction pattern, the crystalline phase was analyzed by the device software (OIM Analysis, Ametek).

#### Wettability

The wettability of the specimens in each surface treatment group (n = 6) was evaluated using a contact angle meter (CA-X; Kyowa Interface Science, Saitama, Japan). Pure water was put into a micro-syringe equipped with the device. A droplet (0.4 μL) of pure water created at the tip of the syringe was contacted to the surface of the specimen, and the syringe was lifted away. The contact angle of the droplet on the surface was measured within 5 s.

#### Hardness and modulus of elasticity

Indentation hardness and modulus of elasticity (E-modulus) were evaluated by a nano-indentation technique using a dynamic ultra-micro hardness tester (DUH-211; Shimadzu, Kyoto, Japan). Half of the specimens (n = 6) prepared for this analysis were subjected to autoclaving under the same conditions as described above, and the remaining 6 specimens were used without autoclaving. The specimen, fixed in the device using metal strips, was loaded via the Berkovich indenter at a rate of 14 mN/s up to 500 mN. After reaching the maximum load, the force was maintained for 15 s and then unloading was performed at the same rate. Based on the load-displacement curve, nano-indentation hardness and E-modulus were calculated using the device software. For the calculation of E-modulus, Poisson’s ratio of 3Y-TZP was assumed to be 0.3, according to the literature [25].

### Bacterial adhesion assay

The 3Y-TZP specimens were ultrasonically cleaned in pure water (5 min), 70% ethanol (5 min), acetone (5 min), and additionally in pure water (5 min). Immediately before the bacterial assay, the specimens were immersed in 2% glutaraldehyde (Denthyde; Nippon Shika Yakuhin, Shimonoseki, Japan) for 1 h followed by washing with sterile water to obtain aseptic specimens.

Two species of oral streptococci, *Streptococcus mitis* JCM 12971 and *S*. *oralis* JCM 12997, were provided by Japan Collection of Microorganisms (Riken BioResource Center, Wako, Japan). Cultures were anaerobically grown using AneroPack (Mitsubishi Gas Chemical Company, Tokyo, Japan) in brain heart infusion (BHI) broth (Becton Dickinson Labware, Franklin Lakes, NJ) at 37°C for 24 h. From this culture, each bacterial suspension was prepared in sterile saline to a concentration of approximately 10^8^ colony-forming units (CFU)/mL.

To create a pellicle on the surface, the specimens were incubated with 500 μL of sterile artificial saliva (phosphate-buffered saline supplemented with 40 μg/mL bovine serum albumin, 0.01 mg/mL α-amylase, 10 μg/mL lysozyme and 850 mg/L mucin) in a well of a 48-well cell culture plate at 37°C for 1 h according to a previous study [26]. Following incubation, the artificial saliva was removed, and the wells were inoculated with 1 mL of BHI broth supplemented with 1% yeast extract (Oxoid, Hampshire, UK) and 100 μL of the bacterial suspension. The plate was then incubated anaerobically at 37°C for 3 and 6 h to simulate early dental plaque formation. The biofilm formed on the specimens was washed twice with saline to remove non-attached bacteria. Then, the remaining bacteria on the biofilm were collected using an enzymatic detachment technique that was based on a previous study with some modifications [27]. Briefly, the specimen was immersed in 300 μL of an enzyme suspension composed of 4 mg/mL type I collagenase (Thermo Fisher Scientific) and 2 mg/mL dispase (Thermo Fisher Scientific) in phosphate-buffered saline. The 48-well plate containing samples was incubated for 2 h under rotation at 200 rpm at 37°C. Subsequently, the specimen and enzyme solution were transferred to 1.5-mL microtubes, and each tube was vortexed for 10 s. The mixture was serially diluted 10-fold in saline, and 10 μL of the dilution was plated onto BHI agar (Oxoid). Agar plates were cultured anaerobically at 37°C for 24 h, followed by colony counting for determination of the number of CFUs per specimen. All tests were performed in six independent assays.

The *S*. *mitis* and *S*. *oralis* biofilms formed on the specimens in the UT and LTD-US-S groups after incubation periods of 3 and 6 h were additionally analyzed using confocal laser scanning microscopy (CLSM), according to a previous study [28]. Briefly, the biofilm was fluorescently stained with a LIVE/DEAD BacLight Bacterial Viability Kit (Thermo Fisher Scientific, Waltham, MA) containing SYTO^®^9 and propidium iodide (PI) for 20 min at room temperature. Both living and dead bacterial cells were stained with SYTO9 (green) while only dead cells were stained with PI (red). The final concentrations of SYTO9 and PI were 10 and 60 μM, respectively. The biofilm was imaged under lasers with an excitation wavelength of 488 nm for SYTO9 and 532 nm for PI using CLSM (TCS-SPE; Leica Microsystems, Wetzlar, Germany) with a 63× water immersion objective lens. CLSM images were obtained at six sites per specimen, where the bacteria attached and proliferated. The biofilm coverage (%) against the observed surface was computed by binarizing the images using ImageJ (National Institutes of Health, Bethesda, MD). The mean value obtained in each specimen was regarded as the representative value. All tests were performed in three independent assays.

### Statistics

Statistical analyses were performed using JMP Pro 13.1.0 software (SAS Institute, Cary NC, USA). Normal distribution of the data was verified using the Shapiro–Wilk test. Since the data for surface roughness after each treatment were not normally distributed, significant differences (p < 0.05) were analyzed using the Steel–Dwass multiple comparison test. The other data could be assumed to have a normal distribution. Thus, significant differences (p < 0.05) were assessed by the Welch’s *t*-test for pairwise comparisons or by the analysis of variance (ANOVA) followed by the Tukey–Kramer HSD test for multiple comparisons. Regarding the bacterial counts, logarithmically transformed CFU/specimen values were subjected to three-way ANOVA to analyze the factors (incubation time, LTD, ultrasonic scaling, and their combinations) influencing the bacterial adhesion on the specimens, followed by the Tukey–Kramer HSD test for multiple comparisons.

## Results

### Surface roughness

**Fig 1:**
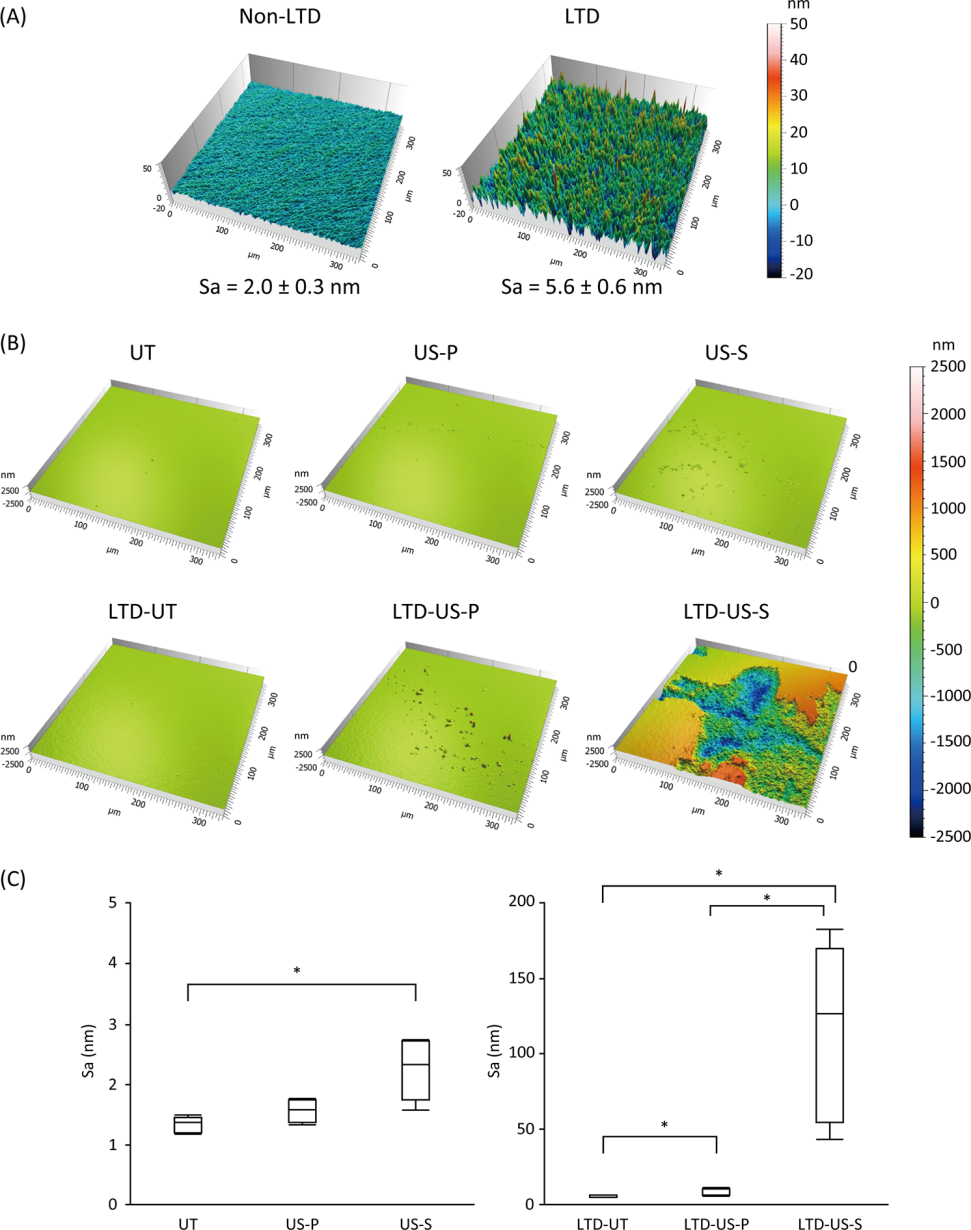
**Representative images of zirconia (3Y-TZP) surface affected by low-temperature degradation (LTD) (A) and ultrasonic scaling in combination with LTD (B), and boxplots for the Sa (arithmetic mean height) of each treatment group.** The surface was analyzed using an optical interferometer with a field of view of 336 × 336 μm. The difference in Sa between non-LTD and LTD specimens shown in (A) was significant (p < 0.01). Ultrasonic scaling significantly increased the surface roughness, especially when LTD was induced and a steel scaler tip was used (LTD-US-S), as shown in (B) and (C). In graph (C), the line within the box represents the median (n = 6), and the bottom and top of the box represent the first and third quartiles, respectively. The maximum and minimum whiskers represent the 1.5 interquartile range. Significant differences (p < 0.05) between the groups are indicated with an asterisk (*). UT: untreated, US-P: ultrasonic scaling using a plastic scaler tip, US-S: ultrasonic scaling using a steel scaler tip.

Surface roughness analysis showed that the autoclaving-induced LTD significantly increased the surface roughness of 3Y-TZP (p < 0.0001, Fig 1A), although the difference in Sa between the groups with or without LTD was only approximately 3.6 nm. In addition, ultrasonic scaling increased the Sa in both non-LTD and LTD groups. Representative images of the surface after ultrasonic scaling are shown in Fig 1B. In the non-LTD group, Sa in the US-S group was significantly higher than that in the UT group (p < 0.05, Fig 1C). In the LTD group, LTD-US-P and LTD-US-S showed significantly higher Sa than LTD-UT (p < 0.05, Fig 1C). In addition, the difference between the LTD-US-P and LTD-US-S groups was significant (p < 0.05, Fig 1C). Thus, LTD-US-S showed the highest Sa of 117 nm. The other surface roughness parameters (i.e., Sq, Sp, Sv, and Sz) also increased after ultrasonic scaling, especially in the LTD group (Table 1).

**Table 1:** Surface roughness parameters of zirconia (3Y-TZP) treated with or without autoclaving-induced low-temperature degradation and ultrasonic scaling.

			Sa (nm)	Sq (nm)	Sp (nm)	Sv (nm)	Sz (nm)
**Non-LTD**	**UT**	Mean	1.4	3.1	282	249	532
		SD	0.1	1.2	125	87	180
	**US-P**	Mean	1.6	3.9	363	254	617
		SD	0.2	0.9	89	63	149
	**US-S**	Mean	2.3	10.6	572	647	1221
		SD	0.5	4.9	232	513	478
	**UT**	Mean	5.4	7.8	466	255	721
**LTD**		SD	0.6	2.2	412	85	495
	**US-P**	Mean	8.4	27.0	1078	751	1520
		SD	1.7	15.6	630	902	796
	**US-S**	Mean	117.1	187.0	1386	1461	2830
		SD	61.4	55.7	715	176	732

Sa: arithmetic mean height, Sq: root mean square height, Sp: maximum peak height, Sv: maximum valley depth, Sz: distance from peak to valley, LTD: low-temperature degradation, UT: untreated, US-P: ultrasonic scaling using a plastic scaler tip, US-S: ultrasonic scaling using a steel scaler tip, SD: standard deviation

### Crystalline structure

**Fig 2:**
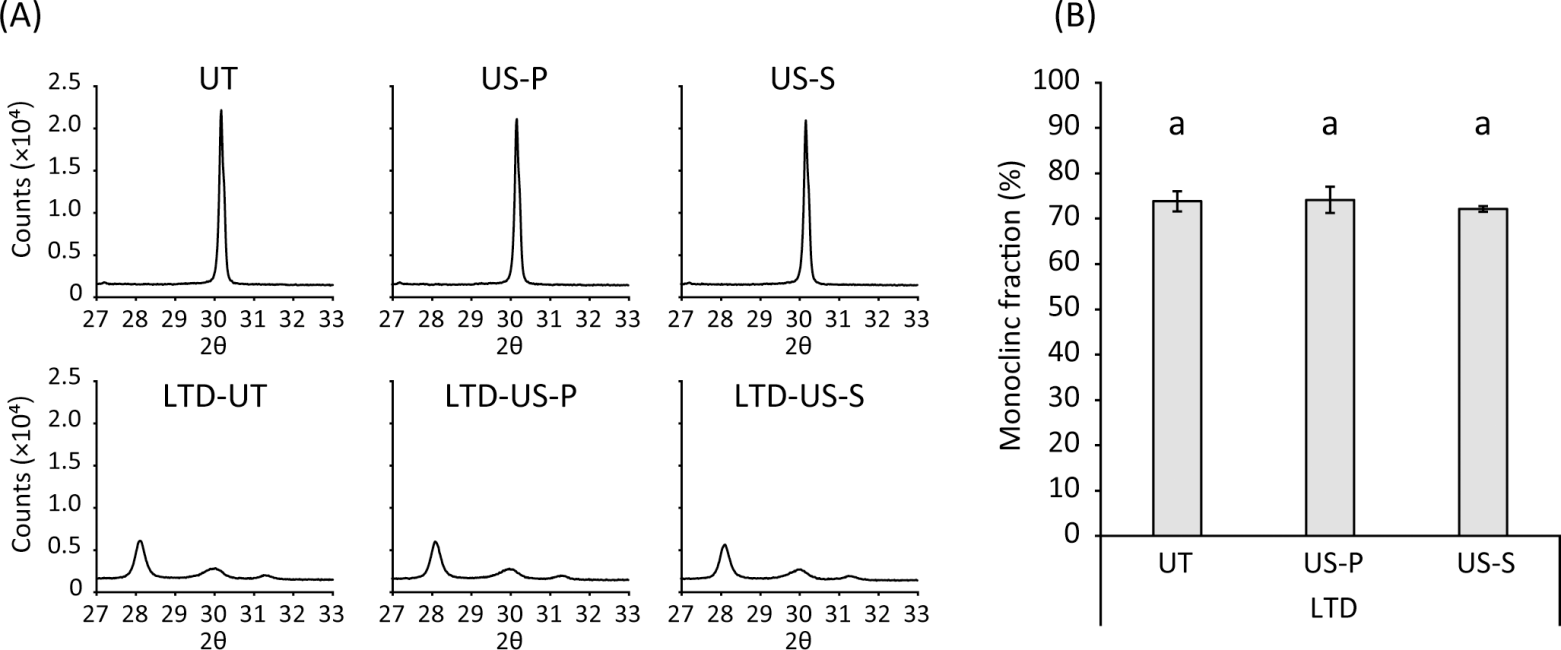
**Representative X-ray diffractograms (A) and a graph showing the quantitative data for the monoclinic fraction generated in zirconia (3Y-TZP) subjected to autoclaving (B).** Peaks at *2θ* = 28.2°, 30.2°, and 31.3° in the diffractograms (A) correspond to *Im (-111)*, *It (101)*, and *Im (111)*, respectively. 3Y-TZP without low-temperature degradation (LTD) did not contain the monoclinic phase. The monoclinic fraction generated by LTD was approximately 70%, and it was not significantly affected by additional ultrasonic scaling (p > 0.05), as shown in (B). The values and error bars indicate the mean and standard deviation, respectively (n = 3). The same letters above the columns in (B) refer to no significant differences (p > 0.05) between groups. UT: untreated, US-P: ultrasonic scaling using a plastic scaler tip, US-S: ultrasonic scaling using a steel scaler tip.

Representative X-ray diffractograms are displayed in Fig 2A. The 3Y-TZP specimens in the UT group showed a peak at *2θ* = 30.2°, corresponding to *It (101)*. Ultrasonic scaling (US-P and US-S) did not affect the peak intensity of *It (101)* and did not generate the peaks of the monoclinic phase. When LTD was induced by autoclaving, peaks of *Im (-111)* and *Im (111)* were observed at *2θ* = 28.2° and 31.3°, respectively (Fig 2A). The monoclinic fraction was calculated to be 73.8% in the LTD-UT group (Fig 2B). Ultrasonic scaling did not affect the peak intensities of *Im (-111)* and *Im (111)*, resulting in monoclinic fractions of 74.2% and 72.1% for the LTD-US-P and LTD-US-S groups, respectively. There were no significant differences in the monoclinic fraction between the LTD-UT, LTD-US-P, and LTD-US-S groups (p > 0.05).

**Fig 3:**
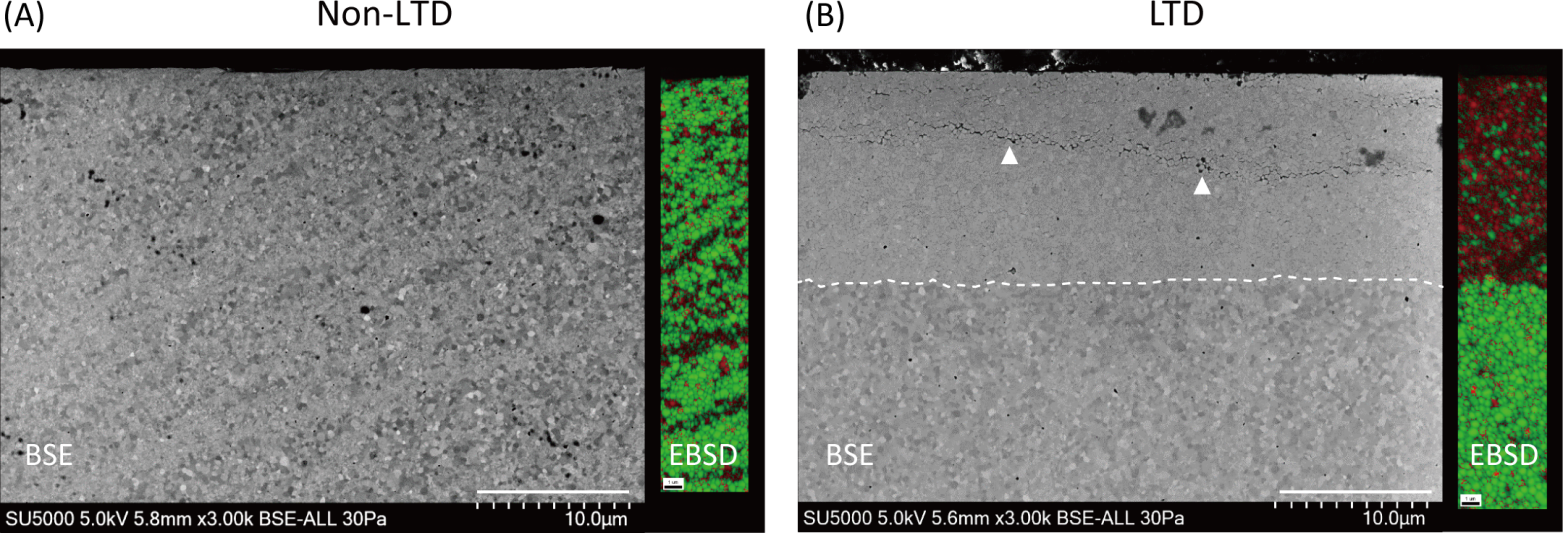
**Representative backscattered electron (BSE) and electron backscattered diffraction (EBSD) images for zirconia (3Y-TZP) without low-temperature degradation (LTD) (A) and after LTD (B).** In the BSE image for the LTD specimen, the transformed layer could be clearly distinguished, which was not seen in the non-LTD specimen. The white dotted line and triangles in (B) indicate the border of the transformed layer and microcracks, respectively. In the EBSD images, the tetragonal and monoclinic phase are shown in green and red, respectively. The EBSD demonstrated that the transformed layer was composed of the monoclinic phase whereas the non-LTD specimen and non-affected area of the LTD specimen consisted of the tetragonal phase. The mean depth of the transformed layer was 16.0 μm (n = 3).

BSE images clearly showed a transformed layer containing microcracks and grain pull-out in the LTD group, whereas no transformed layer was observed in the non-LTD group (Fig 3). The mean depth of the transformed layer was 16.0 μm (SD: 1.7 μm). EBSD analysis additionally demonstrated that the tetragonal grains were homogeneously distributed in the non-LTD group whereas the transformed layer in the LTD group was composed of monoclinic grains (Fig 3).

### Wettability

**Fig 4:**
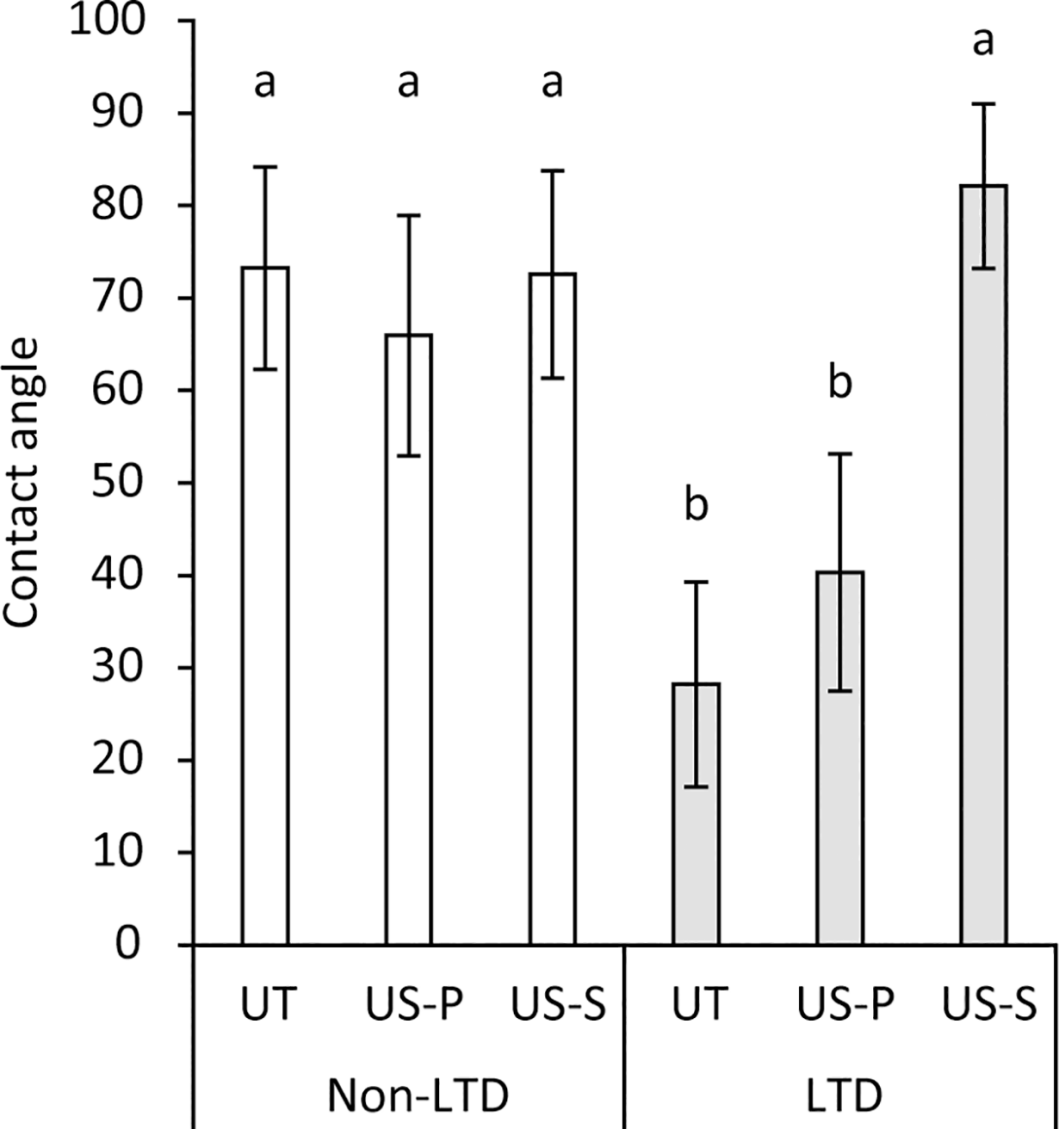
Contact angle of 0.4 μL pure water droplet on the zirconia (3Y-TZP) surfaces subjected to low-temperature degradation (LTD) and ultrasonic scaling. In the non-LTD group, ultrasonic scaling did not significantly affect the contact angle (p > 0.05). When subjected to LTD, the contact angle decreased, but subsequent ultrasonic scaling, especially scaling using a steel scaler tip, increased the contact angle. Values and error bars indicate the mean and standard deviation, respectively (n = 6). Different letters above the columns refer to significant differences (p < 0.01) between groups. UT: untreated, US-P: ultrasonic scaling using a plastic scaler tip, US-S: ultrasonic scaling using a steel scaler tip.

The results of the contact angle measurements are summarized in Fig 4. The surface of 3Y-TZP in the UT group showed a contact angle of 73.2°. The contact angles for the US-P and US-S groups were 66.0° and 72.6°, respectively. There were no significant differences in the contact angles between these three groups (p > 0.05). When LTD was induced by autoclaving, the contact angle significantly decreased, resulting in an angle of 28.2° in the LTD-UT group. Ultrasonic scaling increased the contact angle depending on the type of the scaler tip, yielding angles of 40.3° for the LTD-US-P group and 82.1° for the LTD-US-S group. The difference between the LTD-UT and LTD-US-P groups was not significant (p > 0.05) whereas that between the LTD-UT and LTD-US-S groups was significant (p < 0.01).

### Hardness and modulus of elasticity

**Fig 5:**
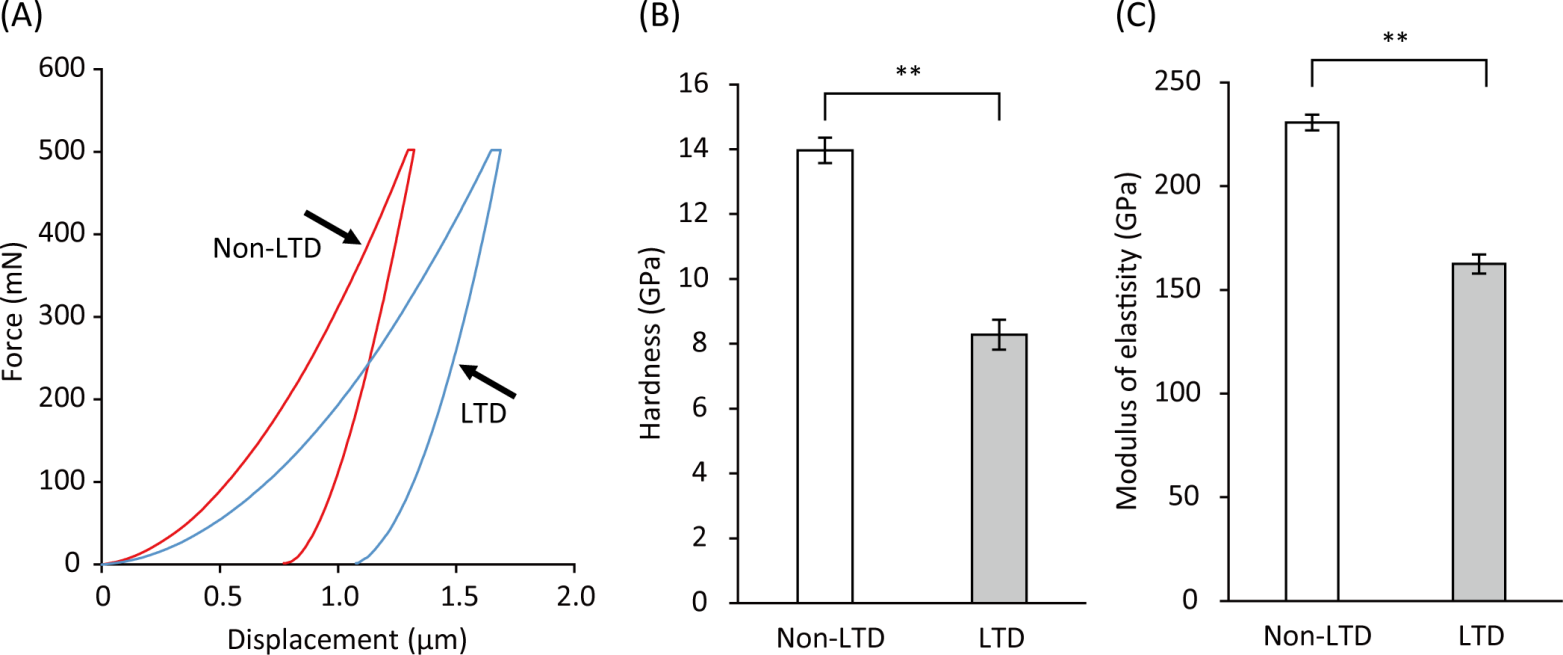
**Representative load-displacement curves (A) and quantitative results for indentation hardness (B) and modulus of elasticity (C) for zirconia (3Y-TZP) with or without low-temperature degradation (LTD).** The load-displacement curve was obtained by a nano-indentation technique with a maximum load of 500 mN. Indentation hardness and modulus of elasticity were calculated based on the load-displacement curve. When LTD was induced, the depth of indentation was deepened compared with that in non-LTD 3Y-TZP, resulting in significantly lower indentation hardness and modulus of elasticity (p < 0.01). Values and bars in (B) and (C) indicate the mean and standard deviation, respectively (n = 6). Significant differences (p < 0.01) between groups are indicated with an asterisk (**).

Representative load-displacement curves for 3Y-TZP specimens in the non-LTD and LTD groups obtained in the nano-indentation analysis are shown in Fig 5A. The specimens in the non-LTD group showed a shallower depth of indentation (displacement) than those in the LTD group. The nano-indentation hardness values calculated on the basis of the load-displacement curves were 13.97 and 8.28 GPa for the non-LTD and LTD groups, respectively (Fig 5B), with a significant difference between the two (p < 0.01). In addition, the E-modulus was also affected by LTD, resulting in values of 230.6 GPa for the non-LTD group and 162.5 GPa for the LTD group (Fig 5C), with a significant difference between the two (p < 0.01).

### Bacterial adhesion assay

**Fig 6:**
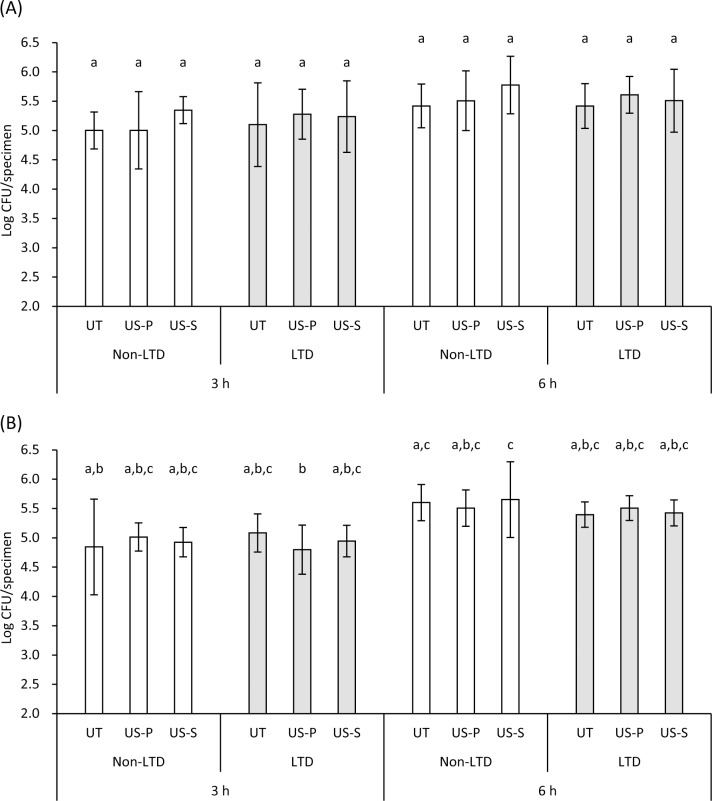
**Viable bacterial counts of *S*. *mitis* (A) and *S*. *oralis* (B) 3 and 6 hours after incubation on zirconia (3Y-TZP) specimens subjected to low-temperature degradation (LTD) and ultrasonic scaling.** The bacterial counts increased in a time-dependent manner whereas LTD and ultrasonic scaling did not significantly affect the bacterial adhesion and proliferation on the specimens (see Table 2). Values and bars indicate the mean and standard deviation, respectively (n = 6). Different letters above the columns refer to significant differences (p < 0.05) between groups. CFU: colony-forming units, UT: untreated, US-P: ultrasonic scaling using a plastic scaler tip, US-S: ultrasonic scaling using a steel scaler tip.

Viable bacterial counts of *S*. *mitis* and *S*. *oralis* after 3 and 6 h of incubation on the treated 3Y-TZP are summarized in Fig 6. The bacterial counts of *S*. *mitis* in the UT group with incubation periods of 3 and 6 h were 5.0 and 5.4 log CFU/specimen, respectively. Similarly, the bacterial counts of *S*. *oralis* in the UT group with incubation periods of 3 and 6 h were 4.8 and 5.6 log CFU/specimen, respectively. Three-way ANOVA revealed that the incubation period significantly affected the viable bacterial counts (Table 2). In contrast, neither LTD and ultrasonic scaling nor their combination significantly affected the viable counts of *S*. *mitis* and *S*. *oralis* on 3Y-TZP specimens (Table 2). Multiple comparison test revealed that there was no significant difference in CFU/specimen of *S*. *mitis* between each group while the differences in CFU/specimen of *S*. *oralis* between some groups with 3-h incubation and those with 6-h incubation were statistically significant (Fig 6).

**Table 2:** Summary table of three-way ANOVA for the factors influencing viable bacterial counts of *S*. *mitis* and *S*. *oralis* on zirconia (3Y-TZP) specimen.

	Source	Sum of squares	df	Mean square	F value	P value
*S*. *mitis*	Time	2.57539	1	2.57539	10.9464	0.0016
	LTD	0.00493	1	0.00493	0.0210	0.8854
	US	0.65309	2	0.32655	1.3880	0.2575
	Time*LTD	0.09315	1	0.09315	0.3959	0.5316
	Time*US	0.01450	2	0.00725	0.0308	0.9697
	LTD*US	0.43587	2	0.21794	0.9263	0.4016
	Time*LTD*US	0.00440	2	0.00220	0.0093	0.9907
	Error	14.11631	60	0.23527		
*S*. *oralis*	Time	6.05001	1	6.05001	38.3425	<0.0001
	LTD	0.07619	1	0.07619	0.4829	0.4898
	US	0.01289	2	0.00644	0.0408	0.9600
	Time*LTD	0.11345	1	0.11345	0.7190	0.3998
	Time*US	0.01799	2	0.00899	0.0570	0.9447
	LTD*US	0.05947	2	0.02973	0.1884	0.8287
	Time*LTD*US	0.34638	2	0.17319	1.0976	0.3403
	Error	9.46732	60	0.15779		

* Time: incubation time, LTD: low-temperature degradation, US: ultrasonic scaling, df: degree of freedom

**Fig 7:**
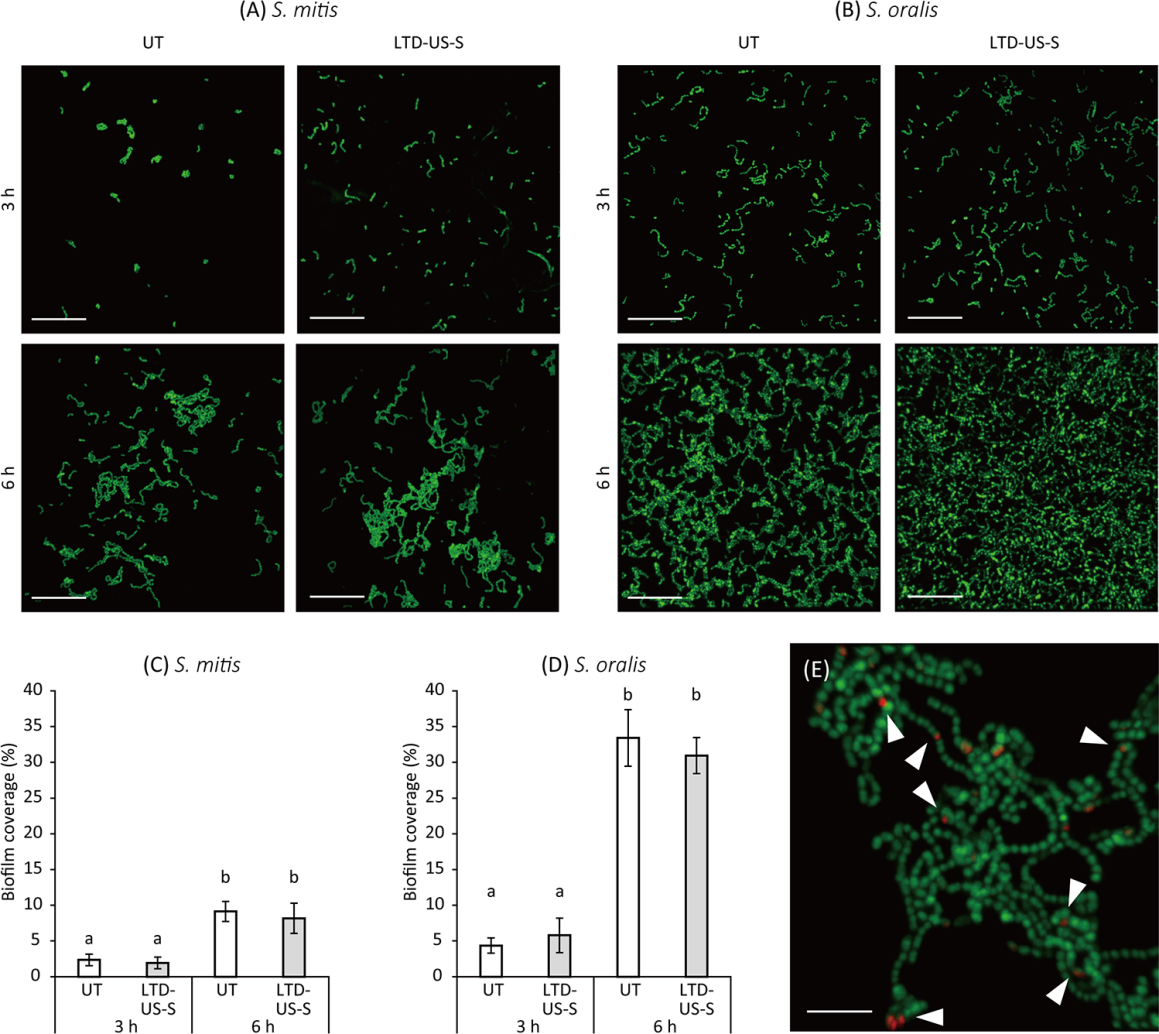
Microscopic analysis of *S*. *mitis* and *S*. *oralis* grown on zirconia (3Y-TZP) specimens subjected to low-temperature degradation (LTD) and ultrasonic scaling (US). (A and B) Representative confocal laser scanning microscope (CLSM) images of *S*. *mitis* and *S*. *oralis*. Living and dead bacterial cells were stained with SYTO9 (green) and propidium iodide (PI) (red), respectively. The merged images were mostly composed of bacterial cells stained with SYTO9. (C and D) Change in biofilm coverage (%) by *S*. *mitis* and *S*. *oralis* on the observed area. For both bacteria, the biofilm coverage significantly increased with time. However, there was no significant difference in the biofilm coverage between the untreated (UT) specimen and the specimen subjected to LTD followed by US using a steel tip (LTD-US-S). (E) Representative CLSM image of *S*. *mitis* after 3 h of incubation obtained at higher magnification. Several bacterial cells were stained with PI as indicated by the white triangles. The scale bars in (A and B) and (E) indicate 20 μm and 5 μm, respectively. Values and bars in (C and D) indicate the mean and standard deviation, respectively (n = 3). Different letters above the columns refer to significant differences (p < 0.01) between groups.

Representative CLSM images of the *S*. *mitis* and *S*. *oralis* grown on the specimens after 3 and 6 h of incubation are shown in Fig 7A and 7B, respectively. Spherical and chained bacterial cells were observed. The biofilm coverage by both *S*. *mitis* and *S*. *oralis* significantly increased with incubation time (p < 0.01, Fig 7C and 7D); however, there was no significant difference between UT and LTD-US-S at each time point (p > 0.05). Most of the bacterial cells were stained with only SYTO9, while a small number of the bacterial cells were also stained with PI (Fig 7E), indicating that the biofilm was mainly composed of viable bacteria.

## Discussion

The present study tested the hypothesis that repeated ultrasonic scaling in combination with LTD would increase the surface roughness of 3Y-TZP, resulting in acceleration of bacterial adhesion on the surface. The results demonstrated that ultrasonic scaling in combination with LTD significantly increased the surface roughness as hypothesized. However, bacterial adhesion was not accelerated by the increased surface roughness. Thus, only the first part of the hypothesis was accepted.

In the present study, ultrasonic scaling was carried out to simulate professional plaque control, which is repeatedly performed as a part of supportive therapy after prosthodontic treatment. Although the frequency of the supportive therapy and the treatment time for each tooth depends on the patient’s specific requirements, it is recommended that supportive therapy, including oral hygiene control, be conducted at least once every 6 months after prosthodontic and periodontal treatment [29, 30]. According to a systematic review [9], the mean time required to treat one tooth using the ultrasonic/sonic scaler during supportive periodontal therapy ranged from 0.35 to 3.90 min. Based on these findings, ultrasonic scaling was performed for 1 min and repeated 10 times, which mimics supportive therapy performed over 5 years. In addition to repeated instrumentation, dental zirconia prostheses may be affected by LTD in a time-dependent manner. Thus, the influence of LTD was evaluated in an accelerated aging test by autoclaving according to ISO 13356 [22]. Regarding the autoclaving time of 100 h, one previous study indicated that autoclaving at 134°C for 1 h theoretically corresponds to 3–4 years *in vivo* [18], but there is controversy regarding the possibility of a significant error in this estimation [31]. A recent study demonstrated that a longer autoclaving time would be required to simulate LTD of 3Y-TZP at 37°C (e.g., 5-h autoclaving would correspond to 2 years *in vivo)* [32]. Thus, an extended autoclaving time was adopted in the present study so that the LTD would sufficiently cover the possible lifetime of the zirconia dental prostheses.

We analyzed the 3D-surface roughness parameters using an optical interferometry technique that is recommended for use in characterizing dental implant surfaces [33]. This analysis enables the evaluation of a selected area instead of a line, as in the case of 2D profilometry. When 3Y-TZP was not autoclaved, the damage caused by ultrasonic scaling to the surface was limited, with an increase in Sa of <1 nm, though the difference between UT and US-S was significant. In the US-S group, micropits instead of scratches were created on the surface, indicating that the surface coarsening was caused by pull-out of grains. Previous studies demonstrated that ultrasonic scaling did not increase the surface roughness of zirconia [12, 34]. However, the treatment time in those studies was much shorter than that used in the present study, which would be the reason for the discrepancy in the findings. When LTD was induced, the surface of 3Y-TZP was roughened even without ultrasonic scaling, which would be a result of volume expansion of the grains, induced by tetragonal to monoclinic phase transformation [35]. This finding is in accordance with a previous study demonstrating that the surface roughness (Sa) of 3Y-TZP subjected to autoclaving increased in a time-dependent manner [36]. Ultrasonic scaling further increased the surface roughness of autoclaved 3Y-TZP. In the LTD-US-P group, the surface contained small peaks instead of pits, indicating that the surface coarsening might be due to the accumulation of debris of the plastic scaler tip. The surface defects created by LTD might allow the debris to firmly attach to the surface, which could not be removed by ultrasonic cleaning in pure water. The reason why the debris accumulated on the autoclaved surface but not on the intact surface is unknown, and as such, is worth studying further. The increase in Sa in the LTD-US-P group was, however, still <10 nm. In contrast, the increase in Sa in the LTD-US-S group was >100 nm. Some areas of the surface were clearly worn-out by the US-S. This is most likely due to the degradation of the surface mechanical properties, including surface hardness, as demonstrated by the nano-indentation analysis.

In line with previous reports [20, 32, 37], autoclaving-induced LTD decreased the hardness of 3Y-TZP as well as the E-modulus. As previously discussed [38], the decrease in the mechanical properties would be due to the presence of microcracks in the transformed layer as observed in the cross-sectional surface analysis, which results in the loss of contact stiffness. In addition, since the transformed layer consisted of approximately 70% monoclinic phase, as demonstrated by XRD and EBSD, stress-induced transformation from the tetragonal to the monoclinic phase may be no longer induced in the layer. Thus, the transformed layer generated by LTD would be more vulnerable to the mechanical damage caused by the US-S. The depth of the transformed layer was about 16.0 μm according to SEM analysis, which was comparable to the values reported in previous studies [19, 20]. In contrast, the Sz (the largest distance from peak to valley) recorded in the LTD-US-S group was still approximately 2.8 μm. Therefore, it is reasonable to assume that the remaining transformed layer of 3Y-TZP in the LTD-US-S group may be more worn-out when ultrasonic scaling is additionally performed using a steel scaler tip.

Bacterial adhesion on the surface of 3Y-TZP subjected to ultrasonic scaling and LTD was evaluated using *S*. *mitis* and *S*. *oralis*, which are known as initial colonizers among the bacteria constituting dental plaque [39, 40]. Since the surface properties of zirconia would affect the early plaque formation rather than its maturation process [41], the adhesion of the initial colonizers was analyzed in the present study. The results demonstrated that the viable counts of both bacterial species on the 3Y-TZP specimen were not affected by the changes in surface properties, such as surface roughness and wettability, caused by ultrasonic scaling and LTD. This finding was further confirmed by CLSM, which showed that there was no significant difference in biofilm coverage on the observed surface between the UT and LTD-US-S groups, whereas the biofilm coverage increased with incubation time. As described above, the surface roughness was significantly affected by ultrasonic scaling and autoclaving, especially by the combination of the two, resulting in an Sa = 117 nm (0.117 μm) in the LTD-US-S group. According to previous studies, the threshold value of surface roughness was proposed to be Ra = 0.2 μm (Ra is arithmetic mean height in 2D profilometry), below which no or only minor influence of the surface topography occurred on bacterial accumulation [42–44]. This may explain why there was no significant difference in the bacterial adhesion between the groups with different surface roughness (Sa = 0.001–0.117 μm) in the present study. The Sa value of 0.117 μm is comparable to or lower than the Ra or Sa values for dental ceramics polished by clinical procedures [45], suggesting that the surface roughness caused by ultrasonic scaling in combination with LTD may be clinically acceptable.

The wettability of 3Y-TZP was also significantly changed by the treatments. When not subjected to autoclaving, the polished surface of 3Y-TZP was hydrophobic as previously reported [11, 46], irrespective of whether ultrasonic scaling was performed (contact angle: approximately 70°). In contrast, the surface affected by LTD was hydrophilic (contact angle: approximately 28°), and additional ultrasonic scaling using a steel scaler tip returned the wettability from hydrophilic to hydrophobic. The hydrophilicity may be due to the increase in the number of polar hydroxyl groups as a result of hydrolysis of the Zr-O-Zr bond [47], and ultrasonic scaling might mechanically remove the surface with the hydroxyl groups, returning the surface to hydrophobic. A previous study demonstrated that the hydrophilic surface (contact angle < 10°) of titanium and titanium-zirconium disks accumulated less dental plaque after 48 h than hydrophobic surfaces, and surface hydrophilicity had a larger influence on dental plaque accumulation than surface roughness [48]. However, the results of the present study showed that there was no significant difference in viable bacterial counts between the LTD-UT group with a hydrophilic surface and the other groups with hydrophobic surfaces. The possible explanation is that the surface in the LTD-UT group with a contact angle of 28° might not be hydrophilic enough to attenuate the bacterial adhesion. In terms of dental plaque accumulation, the influence of LTD on the surface of 3Y-TZP, even if the surface hydrophilicity does not lead to less bacterial accumulation, could be clinically acceptable as long as it does not facilitate bacterial adhesion. However, bacterial adhesion onto the surface of dental materials in the oral cavity is influenced not only by surface roughness and surface hydrophilicity, but also by various bacterial interactions [49]. Since only individual species of bacteria were tested in the present study, future studies using a multi-species bacterial model or an *in vivo* dental plaque model should be conducted to verify the present findings.

Based on the results of the present study, it is suggested that US-S may be acceptable for debridement of 3Y-TZP dental prostheses because its damage to the surface of 3Y-TZP was not severe even in combination with LTD and it did not facilitate bacterial adhesion. In addition, US-S itself did not cause phase transformation, suggesting that it does not affect the mechanical properties of 3Y-TZP related to phase transformation. However, if US-P can clean the surface of the zirconia dental prostheses and remove mineralized deposits sufficiently to prevent secondary infectious dental diseases, US-P may be more favorable than US-S because the former would cause less damage than the latter, especially in aged 3Y-TZP dental prostheses in an oral environment. In addition, US-S may cause chipping of 3Y-TZP dental prostheses at the margin or sharp edges of zirconia FDPs and may result in grayish stains due to wear-out of the steel scaler tip, thereby spoiling the esthetics of the dental prostheses, a factor that was not evaluated in the present study. Therefore, further studies are necessary to address these issues before definitive conclusions can be drawn.

## Supporting information

S1 Dataset Datasets generated and analyzed during the current study. (XLSX)
